# Efficacy of a Computer-Based Learning Program in Children With Developmental Dyscalculia. What Influences Individual Responsiveness?

**DOI:** 10.3389/fpsyg.2020.01115

**Published:** 2020-07-15

**Authors:** Juliane Kohn, Larissa Rauscher, Karin Kucian, Tanja Käser, Anne Wyschkon, Günter Esser, Michael von Aster

**Affiliations:** ^1^Department of Psychology, University of Potsdam, Potsdam, Germany; ^2^Academy of Psychotherapy and Intervention Research, University of Potsdam, Potsdam, Germany; ^3^Department of Child and Adolescent Psychiatry, German Red Cross Hospital, Berlin, Germany; ^4^Center for MR Research, University Children’s Hospital Zürich, Zurich, Switzerland; ^5^Children’s Research Center, University Children’s Hospital Zürich, Zurich, Switzerland; ^6^Ecole Polytechnique Fédérale de Lausanne (EPFL), Lausanne, Switzerland; ^7^Center of School and Mental Rehabilitation, German Red Cross Hospitals, Berlin, Germany

**Keywords:** developmental dyscalculia, mathematics instruction, computer-based training, intelligent tutoring system (ITS), numerical development, evaluative study, primary school

## Abstract

This study presents the evaluation of a computer-based learning program for children with developmental dyscalculia and focuses on factors affecting individual responsiveness. The adaptive training program Calcularis 2.0 has been developed according to current neuro-cognitive theory of numerical cognition. It aims to automatize number representations, supports the formation and access to the mental number line and trains arithmetic operations as well as arithmetic fact knowledge in expanding number ranges. Sixty-seven children with developmental dyscalculia from second to fifth grade (mean age 8.96 years) were randomly assigned to one of two groups (Calcularis group, waiting control group). Training duration comprised a minimum of 42 training sessions à 20 min within a maximum period of 13 weeks. Compared to the waiting control group, children of the Calcularis group demonstrated a higher benefit in arithmetic operations and number line estimation. These improvements were shown to be stable after a 3-months post training interval. In addition, this study examines which predictors accounted for training improvements. Results indicate that this self-directed training was especially beneficial for children with low math anxiety scores and without an additional reading and/or spelling disorder. In conclusion, Calcularis 2.0 supports children with developmental dyscalculia to improve their arithmetical abilities and their mental number line representation. However, it is relevant to further adapt the setting to the individual circumstances.

## Introduction

Solid mathematic skills are not only important for a child’s academic career but are also necessary for numerous situations in every-day life. A weakness in this area cannot only lead to school-related problems but may also affect occupational routes and emotional well-being (Cohen Kadosh et al., 2013). Children with developmental dyscalculia (DD) demonstrate highly diverse performance profiles (Kaufmann and von Aster, 2012) with deficits regarding basic numerical processing, transcoding, counting, arithmetic fact retrieval, basic arithmetic skills, and word problems (e.g., Geary et al., 2007; Kaufmann et al., 2013; Kuhn et al., 2013; Landerl, 2013). Due to different definition and diagnostic criteria, the prevalence of DD in English and German speaking children vary between 1.8 and 5% (Lewis et al., 1994; Esser et al., 2008; Fischbach et al., 2013).

Several studies have demonstrated that targeted interventions can improve different aspects of numerical cognition in children with DD (Dowker, 2004; Bryant et al., 2008; Fuchs et al., 2010). Ise et al. (2012) conducted a meta-analysis concerning the efficacy of different treatment approaches for children with mathematical disabilities and reported a moderate mean effect size (Hedges’ *g* = 0.50) which is comparable to the results of other meta-analyses (Baker et al., 2002; Kroesbergen and van Luit, 2003; Chodura et al., 2015).

Based on empirical evidence different characteristics of effective treatments of children with DD are proposed in the literature. Treatment approaches are considered to be especially effective, when they are adaptive to the child’s learning needs and learning speed (Burns et al., 2010; Moeller et al., 2012). Children with DD benefit from a structured design, hierarchical organization and frequent as well as constant repetition and practice (Fuchs et al., 2008). Reward systems enhance the children’s motivation to solve arithmetic problems (Fuchs et al., 2008; Butterworth and Laurillard, 2010). Since children with DD show diverse deficits, effective training approaches need to address multiple areas of numerical cognition such as basic numerical competencies, conceptual and procedural knowledge and arithmetic fact retrieval (Kaufmann et al., 2003; Dowker, 2007).

During the last years several computer-assisted training systems have been developed.

Those training programs do not aim to replace classic learning therapy interventions conducted by therapists or special need teachers but aim to support the development and automatization of specific cognitive components in the numerical domain (von Aster et al., 2012). In particular, for children with DD a computerized training to enhance numerical cognition offers considerable advantages (Räsänen et al., 2015, 2019). It allows addressing an optimal level of difficulty and learning speed through an individually customized task selection. So called intelligent tutoring systems (ITS) are able to build up an internal image of the learner’s skill and ability profile in form of a “learner model” by studying the child’s actions (von Aster and Lipka, 2018).

Furthermore, a computerized training offers the possibility of immediate feedback about the correctness of a solved task. Direct chronological proximity is central for knowledge acquisition (Krajewski and Ennemoser, 2010). To support this, adaptive computer-based trainings can introduce tasks being slightly challenging and thus may foster the development of new skills. Additionally, the computer represents an attractive learning medium (Kulik and Kulik, 1991; Schoppek and Tullis, 2010) providing intensive training in a stimulating environment (Kullik, 2004). Particularly for children with DD a computerized training provides the possibility of a learning environment detached from competitive performance pressure and peer comparisons in the classroom context and offers a less stressful and socially risk-free setting to explore mathematics (Käser and von Aster, 2013). This is especially important, since the repeated experience of failure may lead to math anxiety or negative attitudes toward the subject or the teacher, which in turn may decrease the achievement potential and learning ability (Ashcraft and Faust, 1994; Kohn et al., 2013).

An overview of different computer-assisted interventions can be found in Räsänen et al. (2015, 2019). Interventions can be differentiated according to their content: training of basic numerical competencies like magnitude comparison, mental number line, or subitizing (e.g., *Number Race* – Wilson et al., 2006; Räsänen et al., 2009; *Rescue Calcularis* – Fischer et al., 2008; Kucian et al., 2011), training of arithmetic fact knowledge (Fuchs et al., 2006) or training of a combination of basic-numerical skills, spatial number representation and (simple) arithmetic facts (Butterworth and Laurillard, 2010; Butterworth et al., 2011; *Calcularis* – Käser et al., 2013a; *Meister Cody* – Kuhn and Holling, 2014).

Different meta-analyses examined the effects of computer-based mathematic instruction, revealing positive effects. For example, Li and Ma (2010) reported an average effect size of 0.28 for computer-based math instruction. They found larger effects for elementary school than for higher education and showed that especially children with learning disabilities benefit from computer-based instruction. Other meta-analyses reported positive (immediate) effects with effect sizes ranging from 0.13 to 0.80 (Kulik, 1994; Fletcher-Flinn and Gravatt, 1995; Kroesbergen and van Luit, 2003; Slavin and Lake, 2008; Ise et al., 2012; Chodura et al., 2015). Only very few studies report additional results concerning long-term effects of computer-based training programs (Chodura et al., 2015). According to recent research (meta-analysis) in secondary schools, training programs with high adaptivity to the individual needs of the user outperformed less adaptive types of tutoring systems (Hillmayr et al., 2017).

Additionally, meta-analyses emphasize that the evaluative studies vary highly with respect to sample size, inclusion criteria (severity of math disorders) and outcome variables which influence quality of research and comparability (Seo and Bryant, 2009; Ise et al., 2012; Chodura et al., 2015). A meta-analysis focusing on interventions for children with math difficulties (Chodura et al., 2015) indicated that in at least half of the identified studies a less stringent criterion than recommended by DSM-5 was used to select the study participants, e.g., a rank below the 26th percentile in a standardized mathematical test.

One important step to gain knowledge about the efficacy of training is to understand which circumstances render computer-based training successful and which factors predict training induced improvement (Räsänen, 2015).

So far only few studies addressed this question. For example, Nemmi et al. (2016) found differentiated effects of a combination of a computer-based number line training (NLT) and a computer-based number working memory training (WMT) for children who differ in working memory capacities as well as in mathematic skills. The authors used four training conditions (NLT/reading, WMT/reading, and NLT/WM and reading). While overall the combined training was most effective, they found significant interactions with baseline scores. For example, children with higher working memory capacity reached higher gains (mathematical ability) through the working memory training compared to the number line training. On the other hand, children with higher math performance at baseline benefited more from the number line training.

Another potential predictor for training induced improvement is the coexistence of a reading/spelling disorder. Powell et al. (2009) analyzed differential effects of tutoring (partly computer-assisted instruction) for third-grade students with math difficulties and with or without co-occurring reading difficulties. The study demonstrated a better responsiveness to fact retrieval tutoring on fact retrieval skills for children without a co-occurring reading disorder. In fact, children with a combined disorder did not benefit from the fact retrieval intervention compared to a no treatment condition. It is assumed that children with co-occurring math and reading disabilities show underlying phonological processing deficits (Hecht et al., 2001; Robinson et al., 2002). Therefore, these children could have more severe or various problems performing arithmetic procedures (e.g., counting strategies) as well as retrieving arithmetic facts (e.g., von Aster, 1994, 2000; Geary et al., 2000). Furthermore, results of studies analyzing differences in working memory indicate that the children with double deficits are outperformed by children exhibiting a math disorder in verbal and visuospatial tasks (*Meta-analysis*, Swanson et al., 2009).

One significant non-cognitive factor influencing math performance that received much attention during the last years is math anxiety. Math anxiety is defined as a negative emotional reaction that is characterized by feelings of tension, apprehension, or even dread that interferes with the manipulation of numbers and the solving of mathematical problems in a wide variety of ordinary life and academic situations (cf. Richardson and Suinn, 1972, p. 551; Ashcraft and Faust, 1994, p. 98).

Previous studies have shown that math anxiety can have an adverse effect on longer-term career choices and professional success (Hembree, 1990; Meece et al., 1990; Ma, 1999). In recent years, there are several studies that illustrate a negative relationship between math anxiety and math performance in early elementary school (Wu et al., 2012; Kohn et al., 2013; Ramirez et al., 2013; Vukovic et al., 2013). It is assumed that math anxious students tend to avoid math-related tasks and situations (Ashcraft and Faust, 1994; Ashcraft et al., 2007). They show less confrontation with mathematic tasks, learn less and as a consequence show reduced achievement scores. In addition, they probably receive more negative feedback which increases in turn math anxiety, contributing to a vicious circle (Krinzinger and Kaufmann, 2006; Dowker et al., 2012; von Aster et al., 2017). Furthermore, it is postulated that math anxiety works as a dual task during task processing that reduces working memory capacity which worsens task performance (Ashcraft and Kirk, 2001; Ashcraft et al., 2007; Ashcraft and Moore, 2009). These assumptions regarding ways of explaining the link between math anxiety and mathematics performance are integrated in the Reciprocal Theory (Carey et al., 2016) that postulates a bidirectional relationship. Supekar et al. (2015) found a significant reduction of math anxiety in students with high math anxiety scores at baseline using a one-to-one math tutoring approach. Beyond these behavioral performance effects, they even report that the brain activity levels in the amygdala of high anxious third-grade children normalizes after the intervention to the level of their peers without math anxiety. Concerning math achievement, both groups (high and low anxious children, grade 3) improved their performance in an arithmetic problem solving task equally, as there was no interaction with math anxiety level. Recent work by Kucian et al. (2018a) has demonstrated that math anxiety is even related to changes in brain structure. Particularly, the volume of the amygdala was reduced, which represents the key area in our brain for negative emotional processing such as fear, stress and anxiety. This growing knowledge underscores the important role of emotional factors in mathematical cognition and emphasizes the far-reaching outcome math anxiety can have.

In summary, there are computer-based programs which have been shown to be effective in enhancing number processing, but most of the available programs provide only limited individual adaptability.

Furthermore, evaluative studies rarely use strict criteria for identification of dyscalculic children (Chodura et al., 2015) and lack to investigate long-term effects. In addition, there are only a few studies that focus on individual differences in response to computer-based math instruction and to our knowledge there seems to be none that addresses dyscalculic children.

Based on the need for research for long-term effects of training effects as well as individual responsiveness in dyscalculic children, the objective of the present study is to evaluate the efficacy of the computer-based training program Calcularis 2.0.

Calcularis 2.0 is based on theoretical neurocognitive foundations of numerical cognition, such as the triple-code model (Dehaene, 1992), the four-step developmental model (von Aster and Shalev, 2007) and further theoretical advancements (see i.e., Kucian and Kaufmann, 2009). In particular, we postulate the existence of a core cognitive magnitude system, which enables even different animal species and also human newborns to discriminate large from small numerosities [that are represented from the right (large) to the left (small) space; Kucian et al., 2018b; Di Giorgio et al., 2019], onto which - in the human neuro-cognitive development – non-symbolic numerical meanings are successively and hierarchically transformed into different symbolic number representations (linguistic number word system, visual Arabic notational system, and spatially oriented mental number line). These growing domain-specific cognitive number representations become neuronally built in different and interconnected areas of the brain and act as tools for learning and performing mental arithmetic and higher mathematical reasoning. They are developmentally dependent on environmentally nurtured sensory-motor and cognitive experiences in the pre- and primary school years, especially on increasing capacities of domain-general cognitive abilities like visual-spatial processing, language, working memory and attentional span.

This process of domain-specific representational transformation, which develops from the early non-symbolic perceptions of numerical magnitude, across the acquisition of culturally transmitted symbolization systems (linguistic, visual Arabic) to a gradually expanding, spatially organized symbolic mental number line may be framed by concepts of general cognitive development like the theory of ‘Representational Redescription (RR)’ postulated by Karmiloff-Smith (1992). RR defines domain-specific cognitive development as (i) being initially constrained by innate predispositions, and (ii) being developmentally formed by the child’s experiences in the physical and social environment, in which early implicit procedural representations are successively redescribed into higher order explicit declarative representations, that are mediated by the domain-general information processing system. Importantly, the RR model has been validated empirically in a large number of studies with typically and atypically developing children, including those with Williams-Syndrome and autism spectrum disorder (Karmiloff-Smith, 1998).

From this theoretical point of view the complex development of number processing and calculation abilities may be disturbed or interrupted at different levels of development and for different etiological reasons relating to different dysfunctional components. Hence, it is not surprising that DD is characterized by highly variable clinical pictures including various possible comorbid conditions (Kaufmann et al., 2011). Therefore, intervention strategies should be highly adaptive to individual demands. Furthermore, they should focus on establishing and automatizing the main representational formats of number magnitudes, including the related transcoding routines, while gradually learning and automatizing arithmetic procedures and fact knowledge. Calcularis 2.0 was developed based on these theoretical assumptions and offers children with DD an approach to deal with different deficits. Calcularis 2.0 is a highly adaptive computer-based training program that combines basic numerical cognition with different number representations and arithmetic abilities.

The present evaluation includes a large sample size of children with DD (using strict criteria for identification). Participants were randomly assigned to the Calcularis group completing a 12-weeks training or to the (waiting) control group receiving no training.

We hypothesize that the Calcularis group shows immediate training effects with medium effect sizes, i.e., demonstrate an increased level of arithmetic performance, basic number processing and spatial number representation compared to the (waiting) control group.

We further predict that there is no stronger increase in performance in domains that were not trained (reading, spelling) compared to the control group, indicating domain specificity of the training. Furthermore, we assess the stability of the training effects after a 3-months interval. We hypothesize that there is an increase or at least a consistent level of performance within the Calcularis group. In addition, we examine the impact of different baseline factors on the individual response to the training. As potentially influencing factors we postulate math anxiety, intellectual ability and the coexistence of a reading/spelling disorder. Specifically, we expect that higher improvement goes along with lower math anxiety scores because we assume that math anxiety could work as an impairing factor for deep engagement with the training content (Ashcraft et al., 2007). Additionally, we assume that children with DD and higher intellectual ability have the potential to reach higher gains (Nemmi et al., 2016) and that children without an additional reading and/or spelling disorder tend to show higher profits (Powell et al., 2009).

## Materials and Methods

### Introduction to Calcularis 2.0

Calcularis 2.0 (von Aster et al., 2016) is a highly adaptive computer-based training program. The program’s theoretical neurocognitive foundation of numerical cognition and development consists of the triple-code model (Dehaene, 1992), the four-step developmental model (von Aster and Shalev, 2007) and further theoretical advancements (Kucian and Kaufmann, 2009).

The program aims to automatize the different number representations, to support the formation and access to the mental number line and to train arithmetic operations as well as arithmetic fact knowledge in expanding number ranges from 0–10 until 0–1,000.

Calcularis consists of different instructional games, which are hierarchically structured according to number ranges and can be further divided into two areas. The first area focuses on different number representations as well as number processing in general. Transcoding between alternative representations (based on triple code model, Dehaene, 1992) is trained and children learn the three principles of number understanding: cardinality, ordinality, and relativity. Games in this area are hierarchically ordered according to the four-step developmental model (von Aster and Shalev, 2007).

The second area covers cognitive operations and procedures with numbers. In this area, children learn the concepts of arithmetic operations and automate them. The difficulty of the tasks is determined by the complexity of the task, the magnitude of numbers involved and the visual aids available to solve the task. In both areas, games can be categorized based on their complexity. Main games are complex games requiring a combination of abilities to solve them. Support games train specific skills and serve as a prerequisite for the main games.

A consistent number notation that accentuates the properties of numbers is used throughout the training program. The notation is encoded by color, form and topology.

Calcularis 2.0 features a user model allowing flexible adaptation based on the internally mapped learning and knowledge profile of the individual child. The mathematical knowledge trained in the game is divided into more than 250 different fine-grained skills [e.g., “writing a (verbally) given number between 0 and 100,” “estimating the quantity of a set of dots,” and “adding to numbers between 0 and 10”]. The skills are hierarchically ordered in a directed acyclic graph called dynamic Bayesian network. Connections between the different skills indicate their relations, i.e., it is for example assumed that being able to add two numbers between 0 and 10 is a prerequisite for adding two numbers between 0 and 100. Each skill is associated with a game. When the child plays the associated game of a skill, the system infers from the correct or wrong answers of the child, how well the child already knows this skill. Since the skills are connected, the system at the same time gains also information about the child’s knowledge of other skills. The representation of the skills as a graph has another big advantage: every child can follow its individual learning path through the network. Some kids will follow the most direct path through the network, training only a subset of the skills. Other kids will have to backtrack and extensively cover the skills in the area they have deficits. Additionally, an error library with typical error patterns allows to provide targeted games for the remediation of specific mistakes. The high adaptivity differentiates Calcularis 2.0 from other computer-based intervention programs that mostly provide only limited adaptability by means of adapting the task difficulty.

**FIGURE 1:**
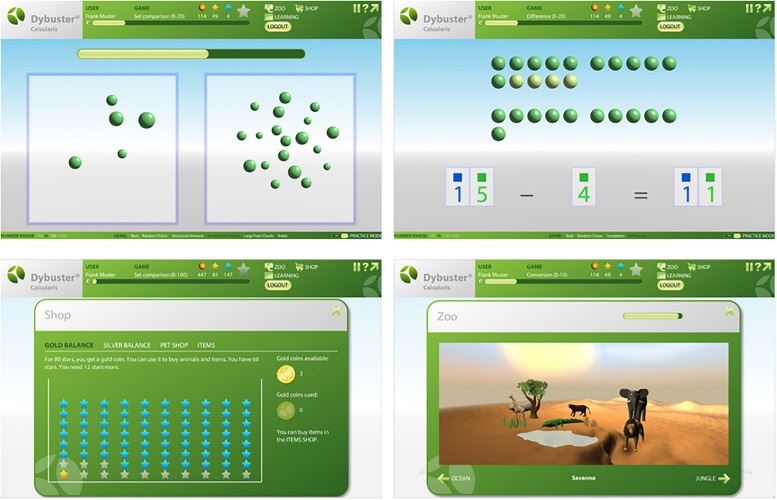
Screenshots from the computer-based training program *Calcularis 2.0*. **(Top)** Left: magnitude comparison non-structured stimuli, right: subtraction with balls. **(Bottom)** Left: reward system – shop to buy and feed animals which can be assigned to various zoo worlds, right: zoo world Savanna.

Calcularis 2.0 represents an extended and modified version of Calcularis (Käser et al., 2012, 2013b). The new version includes additional games to train number and quantity comparisons, subitizing (structured and non-structured stimuli), addition and subtraction based on “concrete” material and multiplication and division (Figure 1, top). Additionally, the number range 0–20 is explicitly modeled. The program includes an interactive avatar guiding the child through the training and explaining the games. Additionally, a reward system (a virtual zoo) reacting to the individual child’s learning progress was implemented to increase the child’s motivation and enhance the enjoyment in learning. The virtual zoo allows children to buy and feed animals which can be assigned to various zoo worlds (Figure 1, bottom). Calcularis, the pre-version of Calcularis 2.0, was evaluated in children with mathematical difficulties as well as in normally achieving children (Käser et al., 2013a; Rauscher et al., 2016). The study results demonstrated that children benefited significantly from the training regarding spatial number representation and subtraction.

### Study Design and Sample

Participants were classified as having DD based on the Diagnostic and Statistical Manual of Mental Disorders (5th ed.; DSM-5) of the American Psychological Association (American Psychiatric Association, 2013).

Criteria for DD were met if a child’s performance in a standardized mathematics test (Rechenfertigkeiten- und Zahlenverarbeitungs-Diagnostikum for the 2nd to 6th grade, RZD 2–6, Jacobs and Petermann, 2005) was 1.5 standard deviations (*T* ≤ 35) below the average in the speed or power component and the IQ-score was within the normal range (*T* ≥ 40) (Basic Diagnostics of specific developmental disorders in elementary school age children, BUEGA, Esser et al., 2008). Children were recruited consecutively via three outpatient clinics as well as via pediatricians in Germany. This approach addressed children with arithmetic problems with and without comorbid disorders. To make sure that enough children fulfill the determined criteria of DD, 107 children were screened.

Children were randomly assigned to the Calcularis group or the control group. Children of the Calcularis group completed a 12 weeks training, while the control group received no training. Children of the control group performed the training between time 2 (*t*_2_) and time 3 (*t*_3_). Children of both groups attended regular schools and visited regular math classes.

Children of the Calcularis group trained with the program 4–5 times per week with training sessions of 20 min after school. Children were assessed before and after the 12-weeks period (*t*_1_/*t*_2_) to evaluate the immediate training effects. To determine the stability of the training effects, children of the Calcularis group were re-assessed after a 3-months-interval (*t*_3_).

Initial diagnostic included the assessment of mathematic competencies (*RZD*, Jacobs and Petermann, 2005) as well as intelligence (*BUEGA*, Esser et al., 2008) and math anxiety (*Math anxiety interview*, *MAI*, Kohn et al., 2013). The pre-/post-/follow-up test diagnostic (*t*_1_/*t*_2/_*t*_3_) for children of both groups included the assessment of arithmetic performance (*Heidelberger Rechentest 1–4*, *HRT*, Haffner et al., 2005), reading and spelling (*BUEGA*, Esser et al., 2008), spatial representation of numbers (number line test 0–100) and basic number processing (basic number processing computer test).

Seventy-two German-speaking children could be included in the study (Calcularis group: *n* = 39, control group: *n* = 33). Only children with at least 42 sessions (corresponds to 70% of the maximum of 60 sessions) of Calcularis within a maximum of 13 weeks of training were included in the analysis. Due to these training-related inclusion criteria as well as other reasons such as illness during the training or test sessions, five children from the Calcularis group were excluded. The final study sample consisted of 67 children between the ages of 7.0–10.11 years attending second to the fifth grade of elementary school. The study population involved more girls (*n* = 49) than boys (*n* = 18), but gender ratio deviated not significantly over the groups.

### Instruments

#### Basic Diagnostics of Specific Developmental Disorders in Elementary School Age Children (BUEGA)

The BUEGA (Esser et al., 2008) served for the assessment of verbal and non-verbal intelligence as well as the performance in reading, spelling, and arithmetic. The internal consistency coefficients determined for each school grade are sufficient to high (α = 0.81 to α = 0.95). The combined score for the reading and spelling performance is the mean value of the scores (standardized *T*-scores) achieved in reading and spelling.

#### Rechenfertigkeiten- und Zahlenverarbeitungs-Diagnostikum for the 2nd to 6th Grade (RZD 2–6)

The RZD 2–6 (Jacobs and Petermann, 2005) is a standardized mathematics test for diagnosing DD. The test assesses basic numerical capacities (e.g., transcoding, counting, number/quantity comparison, and spatial number representation) as well as arithmetic skills (addition, subtraction, multiplication, and division). The test allows for a differentiated assessment of the task performance of the child (power component) and the child’s required time to solve the tasks (speed component). The reliability coefficients of both components (power component: α = 0.89 to α = 0.90; speed component: α = 0.89 to α = 0.92) are sufficient to high.

#### Math Anxiety Interview (MAI)

The MAI (Kohn et al., 2013) served to assess the children’s math anxiety with the help of an anxiety thermometer. The children were asked to rate their intensity of math anxiety in four different situations which were illustrated with pictures. To rate their intensity, they got a thermometer made of cardboard, where they could adjust their fear by manually moving the red column in the thermometer from no anxiety at all or a lot of anxiety. Internal consistency measured using Cronbach’s Alpha is sufficient (α = 0.76).

#### Heidelberger Rechentest 1–4 (HRT)

The scale “arithmetic operations” of the HRT (Haffner et al., 2005) served to assess the children’s arithmetic performance. The scale consists of six subtests (addition, subtraction, multiplication, and division as well as two further subtests with a slightly more complex format: Complete the task by filling in the missing number, e.g., 3 + ? = 5 or put the appropriate relation sign [>, <, =] in the box to show which number [left or right] is larger or if both are equal, e.g., 5 − 1 ? 4).

The HRT is designed as a speed test and specifically addresses computational fluency. For each subtest a score is determined based on the number of correctly solved items within the 2-min time limit This score is converted into a *T*-Score (based on norm values), subsequently the six *T*-Scores are added and in turn converted into a *T*-score for the entire scale.

As an index of reliability, retest reliability was calculated over a 2-week period with medium to high coefficients for the subtests (*r*_*tt*_ = 0.77 to *r*_*tt*_ = 0.89) as well as the over-all scale score (*r*_*tt*_ = 0.93).

#### Number Line Test

As a measure for spatial representation of numbers a number line test from 0 to 100 was administered. Children indicate the location of 20 verbally and visually presented numbers on a number line from 0 to 100. The percent absolute estimation error (PAE) for the target number and the indicated location (estimated number) on the number line was calculated (PAE = | estimated number–target number| /scale of estimates, cf. Siegler and Booth, 2004). In addition, to evaluate the linearity of the spatial representation we calculated the correlation coefficient of linear fit (*R*^2^_lin_) for each child (higher value is associated with better performance). Reliability coefficients estimated for PAE were sufficiently high (α = 0.81 to α = 0.94).

#### Basic Number Processing Computer Test

The subtests *single-digit number comparison*, *two-digit number comparison* and *magnitude comparison* of the computerized test battery of Landerl (2013) served as a measure of basic number processing. In the number comparison subtests (single-digit and two-digit numbers), children were presented with pairs of yellow digits on the computer screen and were asked to select the numerically larger one by pressing the corresponding keyboard button. In the single-digit task, 56 trials with numerical distances from 1 to 8 (36 trials for distance 1–3 and 20 trials for distance 4–8) were presented.

In the 2-digit task 80 trials were presented. To control for a unit-decade-compatibility effect (Nuerk et al., 2004), the influence of differences in the magnitude of decade and unit should be balanced. Therefore, 30 compatible (both decade and unit of one number are larger than decade and unit of the other, so decade and unit comparisons lead to the same response, e.g., 25 36), 30 incompatible (decade and unit comparisons led to different responses, e.g., 25 19), and 20 neutral items (both decades are the same, e.g., 25 29) were presented.

In the magnitude comparison task two quantities of randomly arranged yellow squares (20–72) were presented on the screen and children were supposed to select the numerically larger quantity. Out of the 57 trials there were 27 with a small distance (8–16) and 30 trials with a large distance (17–25).

Reaction times and errors were recorded by the computer. Reliability coefficients estimated for reaction times at each assessment point were high (single-digit: α = 0.95 to α = 0.96, two-digit: α = 0.95 to α = 0.96, magnitude comparison: α = 0.90 to α = 0.94).

The proportions of the correctly solved tasks (accuracy) as well as the individual median reaction times (for correct answers within a range of 200 ms to 10,000 ms) were calculated for each child. According to Landerl (2013) both measures (accuracy and speed of response) were combined into one measure, the inverse efficiency (IE), by dividing the median reaction times by the proportion of correct responses.

### Statistical Analyses

Group differences were analyzed using Analyses of Variance (ANOVA) and Chi-square tests. A series of repeated measures general linear model (GLM) analyses as well as *t*-tests for paired samples were conducted to evaluate training effects between assessment time points (*t*_1_ − *t*_2_) as a within-subject factor and group (Calcularis group/control group) as a between-subject factor. The group x time interaction was the primary effect of interest. Effect sizes are expressed as partial eta squared (η^2^) coefficients. Cohen (1988) postulates that η^2^ values between 0.06 and 0.13 are medium effects and η^2^ values greater than 0.14 are large effects. Correlation analyses and hierarchical regressions were applied to determine the effects of baseline factors on the individual response to the training.

## Results

The analyzed sample consisted of 67 children with developmental dyscalculia. The mean age was 8.96 (*SD* = 0.82) years. Children of the Calcularis group trained with the program for an average training duration of 11.47 (*SD* = 0.93) weeks and completed on average 53.29 (*SD* = 5.45, 42–62) training sessions. Statistical analyses revealed no significant differences between the groups for gender, age, arithmetic/numerical performance or control variables (intelligence, spelling, reading, additional reading, and/or spelling disorder) in the initial diagnostic procedure (*t*_1_) (see Table 1). Criteria for a reading and/or spelling disorder were met if a child’s performance in reading (composite of reading speed and accuracy BUEGA) or spelling (grapheme score BUEGA) was 1.5 standard deviations below the average (*T* ≤ 35).

**TABLE 1:** Demographic and cognitive characteristics [*Mean* (*SD*)] of the Calcularis group (CAL) and the control group (CG) prior to the intervention (*t*_*1*_).

	CAL	CG	Test statistic	*p*
	(*n* = 34)	(*n* = 33)		
Gender (f/m)	26/8	23/10	0.39^d^	0.532
Age (years)	8.94 (0.77)	8.98 (0.88)	−0.22^e^	0.830
Calculation power component^a^ (RZD)	34.20 (7.48)	33.70 (6.41)	0.29^e^	0.770
Calculation speed component^a^ (RZD)	29.94^b^ (3.91)	30.76^c^ (4.35)	−0.71^e^	0.478
Mathematical performance^a^ (BUEGA)	35.79 (7.90)	38.21 (7.61)	−1.28^e^	0.207
Intelligence^a^ (BUEGA)	49.18 (6.90)	48.82 (6.38)	0.22^e^	0.826
Reading and spelling^a^ (BUEGA)	40.04 (8.03)	41.00 (8.23)	−0.48^e^	0.632
Reading and/or spelling disorder	18 (52.9%)	13 (39.4%)	1.24^d^	0.266

*^*a*^T-score, RZD speed component not determinable in case of no correct item in one of the subtests leading to reduced sample sizes, ^*b*^n = 25, ^*c*^n = 27, ^*d*^χ^2^ Score, ^*e*^t-score.*

### Immediate Training Effects

The mean values of the pre- and post-test scores regarding arithmetic performance, basic numerical processing and reading and spelling performance are presented in Table 2.

**TABLE 2:** Training effects (mean values and standard deviations) of the Calcularis group (CAL) and the control group (CG) in arithmetic performance, spatial number representation, basic numeric processing and reading and spelling.

Outcome parameter	Group	*n*	*t*_1_	*t*_2_	Effects	*F*	*p*	η^2^
			*M (SD)*	*M (SD)*				
Arithmetic operations^a^ (HRT)	CAL	34	31.35 (5.07)	34.68 (6.27)	Time	12.64	0.001	0.163
	CG	33	32.88 (6.75)	33.30 (6.78)	Group	0.003	0.958	0.000
					Group × Time	7.57	0.008	0.104
Number line test 0–100 (PAE)^b^	CAL	34	7.87 (3.31)	5.74 (2.56)	Time	7.12	0.010	0.099
	CG	33	8.69 (5.25)	8.30 (4.28)	Group	3.99	0.050	0.058
					Group × Time	3.38	0.070	0.049
Number line test 0–100 (*R*^2^_lin_)	CAL	34	0.86 (0.11)	0.93 (0.07)	Time	7.01	0.010	0.097
	CG	33	0.85 (0.15)	0.85 (0.19)	Group	2.32	0.133	0.034
					Group × Time	5.52	0.022	0.078
1-digit comparison, IES (ms)^c^	CAL	31	954.21 (227.96)	819.22 (171.64)	Time	31.70	0.000	0.342
	CG	32	959.54 (166.39)	877.56 (202.77)	Group	0.50	0.480	0.008
					Group × Time	1.89	0.174	0.030
2-digit comparison, IES (ms)^c^	CAL	30	1868.80 (546.17)	1648.90 (461.76)	Time	5.45	0.023	0.083
	CG	32	1998.53 (599.87)	1891.97 (639.96)	Group	2.18	0.145	0.035
					Group × Time	0.66	0.421	0.011
Quantity comparison, IES (ms)^c^	CAL	32	1086.83 (265.19)	871.53 (194.31)	Time	65.63	0.000	0.514
	CG	32	1085.43 (211.27)	976.21 (189.11)	Group	1.05	0.310	0.017
					Group × Time	7.01	0.010	0.102
Reading and spelling^a^ (BUEGA)	CAL	34	40.04 (8.02)	40.51 (7.98)	Time	0.33	0.566	0.005
	CG	33	41.00 (8.23)	39.86 (8.16)	Group	0.01	0.936	0.000
					Group × Time	1.94	0.168	0.029

*^*a*^T-score, ^*b*^distance (percentage) from correct position, ^*c*^inverse efficiency score.*

#### HRT

The repeated-measures GLM for the HRT “arithmetic operations” demonstrated a significant main effect of time (η^2^ = 0.16), but no main effect of group. The group × time interaction was significant with medium effect size (η^2^ = 0.10), indicating that training progress differed between both groups over time. Children of the Calcularis group demonstrated stronger improvements [*t*(33) = −4.32, *p* < 0.001] than the control group [*t*(32) = −0.59, *p* = 0.559].

#### Number Line Test

The results of the number line test with regard to PAE revealed a significant main effect of time (η^2^ = 0.10). The group × time interaction was not significant. There was no main effect of group.

With regard to linearity, the group × time interaction was significant with moderate effect size (η^2^ = 0.08), demonstrating stronger improvements for the Calcularis group [*t*(33) = −4.33, *p* < 0.001] compared to the CG [*t*(32) = −0.18, *p* = 0.857]. There was a significant main effect of time (η^2^ = 0.10), but no main effect of group.

#### Basic Number Processing Computer Test

The analyses for the 1-digit comparison (IES) indicated a significant main effect of time (η^2^s = 0.342), but no effect of group. The group × time interaction was not significant.

The analyses for the 2-digit comparison indicated a significant main effect of time (η^2^ = 0.083), but no effect of group. The group × time interaction was not significant.

Regarding the IES of the quantity comparison task there was a significant group × time interaction (η^2^ = 0.10). Children of the Calcularis group demonstrated stronger gains than the control group with medium effect size. The significant main effect of time (η^2^ = 0.51) shows that both groups improved with regard to IES but the Calcularis group [*t*(31) = 8.14, *p* < 0.001] outperformed the control group [*t*(31) = 3.63, *p* = 0.001]. No significant main effect of group was found.

#### Reading and Spelling Performance

As a measure of domain specificity, the reading and spelling performance was assessed, and the mean of both measures was used as the dependent variable. The analysis yielded no main effects of time, nor group. The interaction between group x time was not significant for the comparison between the Calcularis and the control group.

To summarize, group × time effects were found for the arithmetic operations (HRT), linearity of the number line and quantity comparison tasks, but not for the score PAE (number line task) and the number comparison tasks, implying that the Calcularis group improved on arithmetic performance (including addition and subtraction), spatial number processing and magnitude comparison.

### Stability of the Training Effects

The analysis of the stability of the training effects (*t*_2_ − *t*_3_) refers only to the Calcularis group since the control group served as a waiting control group and received the computerized training during this interval (*t*_2_ − *t*_3_). The results concerning the stability of the training effects demonstrate that the Calcularis group showed moderate to high correlation coefficients (*r* = 0.59 to *r* = 0.88) for all measures of basic numerical processing and arithmetic competencies. The paired samples *t*-tests revealed no significant results demonstrating stable training effects after a 3-months-interval (see Table 3), with the exception of the number line test 0–100 (*R*^2^_lin_). Children showed reduced scores in linearity (*R*^2^_lin_) while the scores were still significantly higher than at the beginning of the training [*t*_*1*_: *M* = 0.86, *SD* = 0.11, *t*_*3*_: *M* = 0.90, *SD* = 0.12, *t*(31) = −2.14, *p* = 0.041].

**TABLE 3:** Stability of training effects of the Calcularis group in arithmetic performance, spatial number representation, basic numeric processing and reading and spelling (mean values and standard deviations for *t*_2_ and *t*_3_), correlation coefficients *r* and *t*-tests.

Outcome parameter	*n*	*t*_2_	*t*_3_	*Correlation r*	*t-*test	
		*M (SD)*	*M (SD)*		*t*	*p*
Arithmetic operations^a^ (HRT)	32	34.91 (6.39)	35.19 (6.37)	0.77	–0.37	0.716
Number line test 0–100 (PAE)^b^	32	5.76 (2.64)	6.19 (2.70)	0.66	–1.10	0.281
Number line test 0–100 (*R*^2^_lin_)	32	0.93 (0.07)	0.90 (0.12)	0.64	2.43	0.021
1-digit comparison, IES (ms)^c^	30	815.18 (176.44)	791.01 (192.08)	0.68	0.90	0.377
2-digit comparison, IES (ms)^c^	28	1634.47 (474.35)	1558.51 (430.32)	0.78	1.31	0.200
Quantity comparison, IES (ms)^c^	31	868.76 (197.10)	886.93 (229.07)	0.59	–0.52	0.606
Reading and spelling^a^ (BUEGA)	32	40.67 (8.08)	41.05 (9.31)	0.88	–0.48	0.635

*^*a*^T-score, ^*b*^distance (percentage) from correct position, ^*c*^inverse efficiency score.*

### Factors Predicting Training Gain

To investigate whether baseline measures predict individual differences in training improvement, we examined the relation between postulated baseline measures and changes in arithmetic performance as the most curriculum-related criterion (HRT arithmetic operations *t*_2_ minus HRT arithmetic operations *t*_*1*_). Results are presented in Table 4, showing significant negative correlation coefficients between arithmetic improvement and math anxiety (*r* = −0.35, *p* = 0.020) and an additional reading/spelling disorder (*r* = −0.43, *p* = 0.005). Additionally, there was a small correlation coefficient between arithmetic improvement and general intelligence (*t*_*1*_) *r* = 0.25, *p* = 0.074, but no significant correlations between arithmetic improvement and number of sessions or Arithmetic operations (*t*_*1*_).

**TABLE 4:** Correlations among predictor measures (*t*_1_) and gain (*t*_2_ − *t*_1_) (*n* = 34).

	1	2	3	4	5	6
(1) Gain (arithmetic operations, HRT)	–	−0.074	0.254^+^	−0.354*	−0.433**	–0.097
(2) Arithmetic operations (HRT)^a^ (*t*_1_)		–	0.064	−0.236^+^	–0.151	–0.222
(3) Intelligence (BUEGA)^a^ (*t*_1_)			–	0.002	–0.071	0.354*
(4) Math anxiety (MAI) (*t*_1_)				–	0.027	0.142
(5) Reading/spelling disorder (*t*_1_)					–	0.161
(6) Number of sessions						–

^+^p < 0.010, *p < 0.05, **p < 0.01, n = 34, ^*a*^T-score.

To examine which of the baseline measures predicted unique variance in mathematics achievement scores (gain) a hierarchical regression analysis was conducted. Independent variables were added in a stepwise procedure. This method allowed to control for general intelligence (*t*_1_) (step 1), before investigating the unique contribution of the potential predictors in step 2 (additional reading/spelling disorder, *t*_1_) and step 3 (math anxiety, *t*_*1*_) to the variance in arithmetic improvement. Results from this model (see Table 5) demonstrated that an additional reading/spelling disorder explained a significant amount of unique variance in arithmetic improvement [Δ*R*^2^ = 0.17, *F*(1,31) = 7.05, *p* = 0.012]. The negative standardized beta-coefficient as well as the negative correlation coefficient indicated that children with an additional reading/spelling disorder show smaller improvements. Additionally, math anxiety also explains a significant amount of unique variance in arithmetic improvement [Δ*R*^2^ = 0.12, *F*(1,30) = 5.48, *p* = 0.026]. The negative beta weight indicated that children with higher math anxiety show less improvement.

**TABLE 5:** Hierarchical regression analysis for the prediction of gain (arithmetic operations, HRT, *t*_2_ − *t*_1_, *n* = 34).

Variable	*R*^2^	Δ*R*^2^	Δ*F*	*Standardized* β	*t*	*p*
Step 1	0.064		(1,32) = 2.21, *p* = 0.147			
Intelligence *t*_1_				0.254	1.485	0.147
Step 2	0.238	0.173	(1,31) = 7.05, *p* = 0.012			
Intelligence *t*_1_				0.224	1.427	0.163
Reading/spelling disorder *t*_1_				–0.417	–2.656	0.012
Step 3	0.355	0.118	(1,30) = 5.48, *p* = 0.026			
Intelligence *t*_1_				0.226	1.537	0.135
Reading/spelling disorder *t*_1_				–0.408	–2.776	0.009
Math anxiety *t*_1_				–0.343	–2.340	0.026

## Discussion

The aim of the present study was the evaluation of the adaptive computer-based training program *Calcularis 2.0* in a sample of dyscalculic children. Furthermore, factors that predict training improvement were investigated.

### Immediate Training Effects

As expected, compared to the (waiting) control group, the Calcularis group demonstrated larger improvements with moderate effect sizes in a standardized math achievement test (HRT) (*g* = 0.49), in spatial number processing (*g* = 0.55) and magnitude comparison (*g* = 0.44). No training effects were found for reading and spelling performance, hence the presented findings can be interpreted as an indicator for domain specificity of the training.

The HRT is designed as a speed test and specifically addresses arithmetic fluency. It is assumed that the training leads to a higher automation of task processing resulting in faster fact retrieval. Compared to the evaluative studies regarding the previous version *Calcularis 1.0* (Käser et al., 2013a; Rauscher et al., 2016; Kohn et al., 2017) the observed training effects are stronger, whereby it has to be considered that *Calcularis 2.0* includes additional tasks and additional motivational components and that the training interval was prolonged. The medium effect sizes are comparable to other trainings (Kroesbergen and van Luit, 2003; Ise et al., 2012; Chodura et al., 2015) and are satisfactory for a sample of children with severe deficits (participants with DD).

Regarding spatial number processing (number line test) the Calcularis group showed a significant decrease in PAE and a significant increase in linearity, but only the change in linearity was significantly higher than in the control group. These findings are in line with a previous study (Käser et al., 2013a) that analyzed PAE and showed an improvement for the number range 0–100 after a 3-months training period. These results are promising, as the mastering of number line tasks constitutes an important step in the numerical development (von Aster and Shalev, 2007) and provides a tool for solving basic arithmetic. However, it must be taken into account that this improvement on the number line task might not only be due to an improvement of this underlying mental number line. Recent studies indicate that the improvement could also reflect an increasing use of helpful strategies, like using reference points at the number line (Ashcraft and Moore, 2012; Link et al., 2014; Peeters et al., 2016).

With respect to basic number processing no training effects were found for number comparison (1-digit/2-digit), but for magnitude comparison. Compared to the control group, the Calcularis group demonstrated larger improvements with moderate effect size. The low baseline level is one possible explanation of these non-expected results regarding number comparison. Compared to the findings of Landerl (2013) who used the same experimental design, the observed inverse efficiency scores (ms) in our study were lower (i.e., better), providing less room for improvement. Furthermore, both groups (CAL and CG) demonstrated decreased IE-scores that may indicate a test repetition effect, so the additional improvement through the training could possibly not be observed (see Table 2 for 1-digit and 2-digit comparison).

Furthermore, the concept of the training program has to be taken in consideration which balances the training time between the area of number representations and arithmetic operations. Additionally, there is a high variety of skills that are trained in the area of number representations. Therefore, some skills are only trained for a short amount of time or especially at the beginning of the training. As mentioned above children are considered to be already quite proficient in 1-digit comparison and because of the highly adaptive training program, the training sequence of this skill was passed rapidly. However, the result concerning magnitude comparison is promising since faster reaction times in symbolic as well as non-symbolic comparisons are related to higher calculation fluency (for detailed review see De Smedt et al., 2013). Of course, it has to be pointed out that this relationship should rather be interpreted bidirectionally and not causally.

### Stability of the Training Effects

Children were re-assessed after a 3-months interval to determine the stability of the training effects. Regarding all measures of basic numerical processing and arithmetic competencies results demonstrated stable performance scores with moderate to high correlation coefficients that indicates that the children keep their relative position. The performance improvements of the intervention (*t*_1_ − *t*_2_) were shown to be stable after a 3-months-interval (*t*_2_ − *t*_3_). Only the linearity results (*R*^2^_lin_) showed a significant decrease (*t*_2_ − *t*_3_), although the scores were still significantly higher than at the beginning of the training. It has to be mentioned that a comparison to a group without any intervention from *t*_*1*_ to *t*_*3*_ is missing because we were unable to include a waiting control group over 6 months due to ethical reasons. Therefore, we were not able to control for developmental effects and the results provide merely indirect evidence for stable training effects.

The found follow-up effects are comparable to Fischer et al. (2008) and even better than in a former study evaluating Calcularis 1.0 (Kohn et al., 2017). Furthermore, the results support the assumption that a prolonged training duration (12 weeks in Calcularis 2.0 instead of 6 weeks in Calcularis 1.0) contributes to more robust effects.

### Factors Predicting Training Improvement

A hierarchical regression analysis indicates that dyscalculic children without an additional reading/spelling disorder as well as those with low math anxiety scores show higher improvement scores.

It is assumed that children suffering from math anxiety tend to avoid math-related tasks (Ashcraft et al., 2007). We therefore suppose that throughout the training high anxious children tended to confront themselves less with the offered tasks, tended to demonstrate less elaborated processing of the content, and tended to show more off-task behavior.

Therefore, they did not improve their achievement scores as much as their non-anxious peers. This assumption could be integrated in the debilitating anxiety model (Carey et al., 2016). As there were no significant differences between low anxious (*n* = 16, *M* = 31.87, *SD* = 4.33) and high anxious children (*n* = 17, *M* = 30.29, *SD* = 3.80) with respect to arithmetic performance at baseline [*t*(31) = 1.12, *p* = 0.273], the hypothesis that math anxiety inhibits a significant improvement should be verified in further analyses. A first look at the number of training sessions indicated that there is no easy answer to this question. There was no significant difference [*t*(31) = −1.16, *p* = 0.257] between high anxious (*n* = 16, *M* = 54.13, *SD* = 5.39) and low anxious children (*n* = 17, *M* = 52.00, *SD* = 5.17) concerning the number of training sessions. Accordingly, the assumption of a different training behavior must be analyzed based on the log data of each child. Due to the focus of this paper these questions should be elaborated in detail in a subsequent study. Before doing so, a theoretical and methodological clarification of the construct “off-task behavior” is necessary, which affects various aspects of the training behavior.

In line with previous research by Powell et al. (2009) we found a better responsiveness to the training for children without an additional reading/spelling disorder. It is assumed that children with a comorbid dyslexia show underlying phonological processing deficits (Hecht et al., 2001; Robinson et al., 2002) and greater deficits in verbal and visuospatial working memory (Swanson et al., 2009). Therefore, children with comorbid dyslexia could have additional problems that could not be addressed successfully in the 12-weeks-training.

Concerning the predictor of intellectual ability at baseline, the result was less substantial. There seemed to be a trend that DD-children with higher intelligence scores showed higher improvement scores. That would be in line with the results presented by Nemmi et al. (2016). In contrast to this study, initial arithmetic performance (*t*_*1*_) did not predict individual responsiveness. That could be attributed to the fact that only children with DD were considered in the present study.

### Limitations and Further Research Indications

When interpreting the findings, some methodological limitations must be considered.

First, the present study design includes the comparison to an untrained control group whereas comparisons to groups receiving alternative trainings are missing. The implementation of an untrained waiting control group allows the delineation of specific training effects to developmental and schooling effects. However, it has to be questioned whether these severely affected children in the untreated group actually did not receive additional support during the waiting period. Factors such as increased parental assistance in math exercises or a stronger response by teachers, as well as expectation effects (in the sense of a placebo effect) are conceivable. An alternative systematic treatment would have strengthened the findings and is therefore planned in future intervention studies.

Second, a high external validity of the clinical sample was required, leading to a high amount of comorbidities such as dyslexia and probably attention deficit hyperactivity disorders (Auerbach et al., 2008; Fischbach et al., 2010).

As this study and Rauscher et al. (2017) show that comorbid dyslexia can influence the results it is absolutely necessary to use a design with a larger sample that enables to compare children with single and combined deficits to replicate the promising effects. Including a larger sample in future training studies would also offer a higher statistical power and allow for a deeper analysis of the differential efficacy as well as for essential replications.

Although a training program focusing on a broad range of mathematical skills and showing a high degree of individualization seems beneficial, it also poses challenges for the evaluation. First, training a variety of skills shortens the training time of each specific skill and thus leads to smaller training effects as mentioned above.

Second, due to the high adaptability of the program, each child pursues a different training trajectory. Since it is not obvious, which aspects of the training lead to which performance improvement, modular tests or deeper analyses of the individual pathways could be beneficial.

## Conclusion

This study demonstrates that the adaptive training program *Calcularis 2.0* can be used effectively to support dyscalculic children in their numerical achievement. The results showed that even after a rather short training period of 12 weeks, solid training effects with regard to arithmetic and spatial number representation could be achieved. Results indicate that especially math anxiety and a co-occurring reading and/or spelling disorder were significant predictors for individual responsiveness to this training. The training effects were shown to be stable after a 3-months-interval.

In practice, *Calcularis 2.0* can be used individually as well as in a group or class setting as a beneficial enhancement of learning intervention and math lessons. Based on the results of this evaluation and former results (Käser et al., 2013a), a training period of at least 3 months with a training frequency of 3–4 training sessions per week is recommended. The children can work on their own, without performance pressure and frightening peer comparisons. It is important to highlight that *Calcularis 2.0* aims not to be a substitute for teachers, since a positive learning development is created by educational skills, methodical knowledge and an encouraging teacher–student relationship. Especially for children with high math anxiety it should be considered that a one-to-one tutoring might be more effective and might address the individual experiences of fear in the former learning history to help to recode and overcome these internal representations. However, it also seems possible to develop and include elements for the detection of emotional states and for an according cognitive behavioral intervention into future learning environments (O’Neill and Gillespie, 2014).

## Data Availability Statement

The datasets generated for this study are available on request to the corresponding author.

## Ethics Statement

The study involving human participants was reviewed and approved by the Ethics Committee of the University of Potsdam. Written informed consent to participate in this study was provided by the participants’ legal guardian/next of kin.

## Author Contributions

JK: conceptualization of the study idea and design, organization of the database, statistical analysis and interpretation of the data, and preparation and revision of the manuscript. LR: conceptualization of the study idea and design, organization of the database, collection and interpretation of the data, and preparation and revision of the manuscript. TK: conceptualization of study idea, writing sections of the manuscript, and revision of the manuscript. KK, AW, and GE: conceptualization of the study design and revision of the manuscript. MA: conceptualization of the study idea and design, interpretation of the data, writing sections of the manuscript, and revision of the manuscript. All authors have contributed and approved the final manuscript.

## Conflict of Interest

The authors declare that the research was conducted in the absence of any commercial or financial relationships that could be construed as a potential conflict of interest.
